# The mitochondrial ATP-dependent potassium channel (mitoK_ATP_) controls skeletal muscle structure and function

**DOI:** 10.1038/s41419-024-06426-x

**Published:** 2024-01-17

**Authors:** Giulia Di Marco, Gaia Gherardi, Agnese De Mario, Ilaria Piazza, Martina Baraldo, Andrea Mattarei, Bert Blaauw, Rosario Rizzuto, Diego De Stefani, Cristina Mammucari

**Affiliations:** 1 Department of Biomedical Sciences, University of Padova, Padova, Italy; 2 Biozentrum, University of Basel, Basel, Switzerland; 3 Department of Pharmaceutical and Pharmacological Sciences, University of Padova, Padova, Italy; 4 Venetian Institute of Molecular Medicine, Padova, Italy; 5 Myology Center (CIR-Myo), University of Padova, Padova, Italy

**Keywords:** Mitochondria, Ion channel signalling

## Abstract

MitoK_ATP_ is a channel of the inner mitochondrial membrane that controls mitochondrial K^+^ influx according to ATP availability. Recently, the genes encoding the pore-forming (MITOK) and the regulatory ATP-sensitive (MITOSUR) subunits of mitoK_ATP_ were identified, allowing the genetic manipulation of the channel. Here, we analyzed the role of mitoK_ATP_ in determining skeletal muscle structure and activity. *Mitok*^−/−^ muscles were characterized by mitochondrial cristae remodeling and defective oxidative metabolism, with consequent impairment of exercise performance and altered response to damaging muscle contractions. On the other hand, constitutive mitochondrial K^+^ influx by MITOK overexpression in the skeletal muscle triggered overt mitochondrial dysfunction and energy default, increased protein polyubiquitination, aberrant autophagy flux, and induction of a stress response program. MITOK overexpressing muscles were therefore severely atrophic. Thus, the proper modulation of mitoK_ATP_ activity is required for the maintenance of skeletal muscle homeostasis and function.

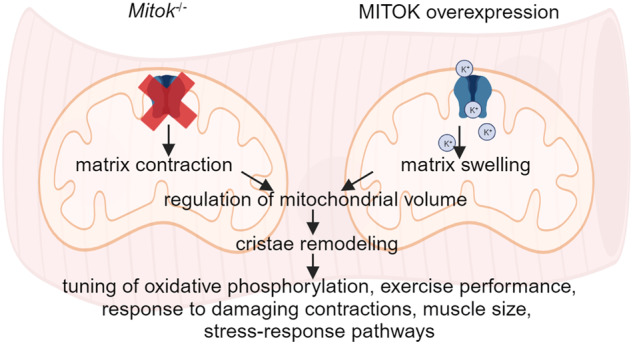

## Introduction

Within mitochondria, dynamic regulation of K^+^ fluxes warrants structural and functional organelle integrity. Despite the huge driving force of the negative membrane potential across the inner mitochondrial membrane (IMM), in physiological conditions the [K^+^] inside and outside mitochondria is similar, due to the low activity of K^+^ channels coupled with the activity of the electroneutral K^+^/H^+^ antiporter. Increased mitochondrial K^+^ influx is accompanied by osmotically obligated water entering through mitochondrial aquaporins, resulting in matrix swelling. By controlling matrix volume, K^+^ cycle contributes to the coupling between respiration and ATP synthesis and to the maintenance of both the structure and the function of the organelle [1]. Thus, K^+^ homeostasis is thought to play a major role in the regulation of mitochondrial activity, including reactive oxygen species (ROS) production, with implications in cell fate and pathophysiological conditions [2]. This is particularly relevant in the response to ischemic/reperfusion injury, which is due to the sudden increase in reactive oxygen species (ROS) production once O_2_ supply is restored. In these settings, the pharmacological opening of mitochondrial K^+^ channels exerts a protective effect [3, 4]. Despite the uncertainty on the precise mechanism, mitochondrial uncoupling preventing ROS generation during reperfusion may be involved.

Mitochondrial K^+^ fluxes must be tightly controlled. High intracellular [K^+^] indeed potentially represents a serious threat for mitochondrial integrity, since excessive K^+^ entry in mitochondria can quickly dissipate the large electrochemical gradient with severe consequences at both organelle and cellular level. Despite this, multiple K^+^ channels are located at the IMM, with different structure, molecular composition, and biochemical features. The need for this redundancy and the specific roles of individual channels are still unclear, but emerging evidence indicates that each of them is controlled by different physiological stimuli, allowing a fine-tuned modulation of the mitochondrial function [1].

Our current understanding of the functions and roles of mitochondrial K^+^ channels was historically limited by several constraints. The most evident one is the lack of biochemical information since the molecular identity of many mitochondrial K^+^ channels is unknown. In other cases where molecular information is available, the same K^+^ channel has been shown to be located at both plasma and inner mitochondrial membranes, but the sorting mechanism remains a mystery. This promiscuous localization prevents the direct investigation of the specific contribution of mitochondrial versus plasma membrane K^+^ channels to organelle and cell physiology. As a consequence, until recently the comprehension of mitochondrial K^+^ fluxes has been largely based on pharmacological approaches that lack specificity and proper genetic validation. This gridlock has been recently solved, at least in part, by the recognition of a K^+^ channel specifically located at the IMM, i.e. the mitochondrial ATP-sensitive K^+^ channel (mitoK_ATP_). The molecular identification of the mitoK_ATP_ channel showed that the assembly of the pore-forming subunit (MITOK) with a regulatory (MITOSUR) subunit is able to mediate ATP-dependent mitochondrial K^+^ entry [5]. *MITOK* ablation causes instability of the mitochondrial membrane potential, widening of the intracristae space and impaired oxidative phosphorylation. Ex vivo, in an ischemia/reperfusion model, *MITOK* deletion suppresses cardioprotection elicited by preconditioning induced by diazoxide, an opener of the mitoK_ATP_ channel. On the opposite side, MITOK overexpression in cell lines triggers mitochondrial fragmentation, swelling, and a drop in mitochondrial membrane potential.

Concerning the skeletal muscle, until recently it was not even clear whether mitoK_ATP_ channels were expressed or not. The first indications were provided by pharmacological modulation of the channel in isolated rat skeletal muscle mitochondria. Those organelles responded to potassium channel openers (KCOs) in terms of increased mitochondrial swelling, oxygen consumption and depolarization, and these effects were counteracted by the addition of K_ATP_ channel inhibitors [6], indicating the existence of mitoK_ATP_ in skeletal muscle, although with different pharmacological properties compared to other tissues. As for respiration, experiments in chicken skeletal muscle mitochondria recorded an inhibition rather than activation by KCOs [7], indicating that different fibre types and metabolic properties could impinge on these responses. Additionally, in adult mouse skeletal muscle bundles, diazoxide reduced muscle fatigue, i.e. the decrease in muscle tension upon repetitive electrical stimulation. The effects of mitoK_ATP_ opening were prevented by 5-HD, a blocker of the channel [8]. In chick muscles, again some differences were observed. In line with the effects detected in the mouse muscles, diazoxide increased post-fatigue tension, but no effect was detected by the addition of 5-HD [9]. Also in this case, the different muscle models (slow versus fast) and the different fatigue protocols may have played a role.

Considering the pivotal role of mitochondrial ion fluxes in the control of skeletal muscle structure and function, here we analysed the consequence of the genetic manipulation of mitoK_ATP_ activity in the mouse skeletal muscle, by deleting or overexpressing MITOK, respectively. Structural and functional assays, corroborated by metabolomics data, indicate a primary role of mitochondrial K^+^ influx to maintain organelle homeostasis, essential for optimal skeletal muscle activity. On the other hand, MITOK overexpression offers a genetic tool, an alternative to protonophore agents, to dissect the outcomes of mitochondrial depolarization and uncoupling. In the skeletal muscle, uncontrolled potassium entry triggers mitochondria dysfunction and energy crisis. Polyubiquitinated proteins accumulate, the autophagic flux is blocked, and a cellular stress response program is induced determining muscle atrophy, despite the persistent activation of the IGF1/AKT1 pathway. Altogether, the genetic manipulation of the mitoK_ATP_ channel uncovers the role of mitochondrial K^+^ fluxes in skeletal muscle structure and function.

## Results

### *Mitok* deletion in skeletal muscle alters mitochondria cristae shape, metabolite profile and respiration

**Fig. 1:**
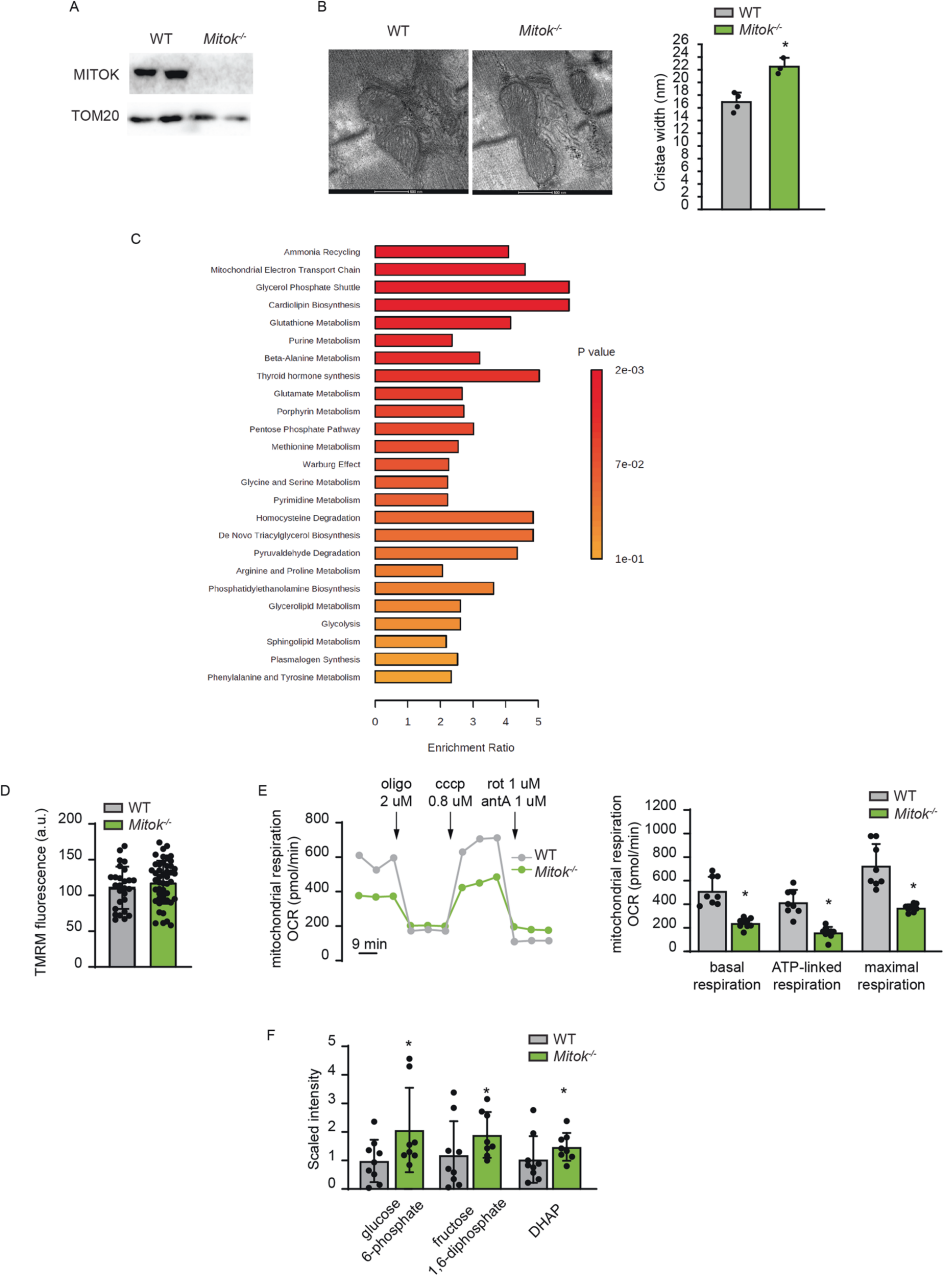
*Mitok* deletion in skeletal muscle alters mitochondria cristae shape, metabolite profile and respiration. **A** Western blot analysis demonstrated efficient *Mitok* deletion in TA muscles. TOM20 was used as protein-loading control. **B** *Mitok*^*−/−*^ muscle mitochondria had enlarged cristae width. On the left: representative TEM images. On the right: quantification. *p < 0.05, t test (two-tailed, unpaired) of 4 WT and 3 *Mitok*^*−/−*^ muscles, respectively. Data are presented as mean ± SD. **C** Untargeted metabolomics analysis of WT and *Mitok*^*−/−*^ muscles. Metabolites set enrichment analysis revealed decreased phosphatidylcoline, phosphatidylethanolamine, dihydrosphingomyelins, and sphyngomyelins pathways in *Mitok*^*−/−*^ muscles. Welch’s two-sample t test of nine animals per condition. **D** FDB fibres were loaded with TMRM and Δψm was measured. t test (two-tailed, unpaired) of at least 25 fibres per condition. Data are presented as mean ± SD. **E** OCR measurements indicated reduced respiratory capacity in *Mitok*^*−/−*^ FDB myofibres compared to controls. On the left: representative traces. On the right: quantification. To calculate basal and maximal respiration, non-mitochondrial O_2_ consumption was subtracted from absolute values. ATP-linked respiration was calculated as the difference between basal and oligomycin-insensitive O_2_ consumption. Data are normalized on mean Calcein fluorescence. *p < 0.05, t test (two-tailed, unpaired) of ten samples per condition. Data are presented as mean ± SD. **F** Untargeted metabolomics analysis of WT and *Mitok*^*−/−*^ muscles. Metabolites set enrichment analysis revealed increased levels of glucose 6-phosphate, fructose 1,6-diphosphate, and DHAP in *Mitok*^*−/−*^ muscles. Welch’s two-sample t test of nine WT and eight *Mitok*^*−/−*^ animals. Data are presented as mean ± SD.

Previous work demonstrated that *MITOK* deletion in HeLa cells causes the accumulation of aberrant mitochondria characterized by widening of the intra-cristae space and decreased respiration. Mitochondrial membrane potential (ΔΨm) is overall intact, but unstable, as shown by the fact that mitochondria undergo asynchronous, rapid and transient depolarizations (i.e. mitochondrial ‘flickering’ or ‘flashes) [5]. Thus, we wondered whether the deletion of *Mitok* triggers similar effects in the skeletal muscle and if muscle function is consequently affected. To this aim, we took advantage of the *Mitok*^*−/−*^ mouse, whose heart was characterized by a slight increase in sensitivity to I/R injury and loss of pharmacological preconditioning by the mitoK_ATP_ opener diazoxide [5]. Western blot analyses confirmed the absence of MITOK protein in the skeletal muscle of *Mitok*^*−/−*^ mouse (Fig. 1A, S4). Skeletal muscle mitochondria of *Mitok*^*−/−*^ animals were characterized by increased cristae width (Fig. 1B), indicating that the hallmarks of MITOK deficiency are conserved in post-mitotic tissues.

To get further insights into the metabolic consequences of defective mitochondrial K^+^ homeostasis, we performed an unbiased metabolomics analysis of 662 compounds in *Mitok*^*−/−*^ muscles compared to WT counterparts (Fig. 1C). *Mitok*^*−/−*^ muscles were characterized by decreased levels of phospholipids, including cytidine 5’-monophosphate, phosphatidylcholines, phosphatidylethanolamines, phosphatidylglycerols, sphingolipids, and phosphatidylinositols, suggesting decreased phospholipid synthesis and/or increased phospholipid breakdown. Notably, phosphatidylglycerols are the main component of the mitochondria-specific lipid cardiolipin and have important roles in membrane remodeling and stress responses [10], suggesting these data may reflect *Mitok*^*−/−*^-induced decreased cardiolipin/mitochondrial biogenesis and/or membrane remodeling. Also, the sphingolipids synthesis intermediates sphinganine and sphingadienine were significantly decreased along with several ceramides, sphingomyelins, and sphingosine in MITOK-deficient muscles relative to controls, suggesting decreased sphingolipid and ceramides synthesis. In addition, cholesterol showed significant decreases in MITOK-deficient muscle. Collectively, the reduced levels of phospholipids, sphingolipids, and cholesterol suggest large-scale remodeling of muscle cell membranes in response to MITOK ablation, in accordance with EM data.

Next, we wished to further characterize the consequence of defective mitochondrial K^+^ homeostasis, by measuring mitochondrial function parameters, including ΔΨm, mitochondrial Ca^2+^ uptake, and oxygen consumption rate (OCR). Deletion of MITOK did not affect the ΔΨm (Fig. 1D) or mitochondrial Ca^2+^ uptake (Fig. S1A-B), while basal, ATP-linked, and maximal respiration were all decreased in myofibres with impaired mitochondrial K^+^ flux (Fig. 1E).

In addition, according to the metabolite profile, *Mitok*^*−/−*^ muscles were characterized by an increase in glucose-6-phosphate that may reflect an increase in hexokinase activity. Moreover, glycolytic intermediates were elevated, including fructose 1,6-diphosphate/glucose 1,6-diphosphate and dihyroxyacetone phosphate (DHAP), suggesting increased glycolysis (Fig. 1F). Thus, defective mitoK_ATP_ activity alters membrane structure, triggers mitochondria cristae widening, and reduces oxidative metabolism in skeletal muscle.

### *Mitok* deletion triggers exercise intolerance and susceptibility to damaging muscle contractions

The above observations, pointing to defective mitochondrial membrane remodelling and oxidative metabolism, lead us to verify the consequences of *Mitok* deletion on muscle structure and activity.

**Fig. 2:**
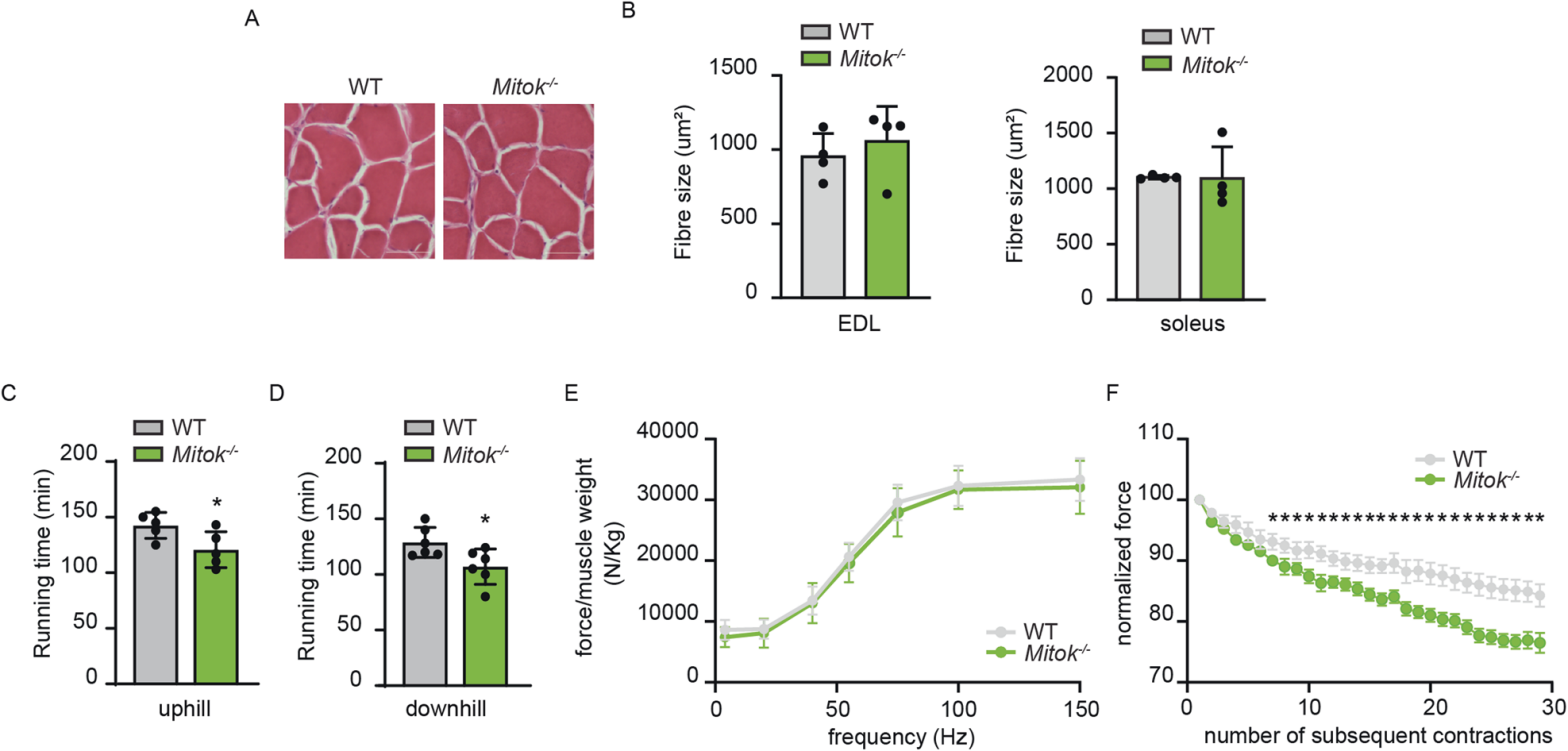
*Mitok* deletion triggers exercise intolerance and susceptibility to damaging muscle contractions. **A** *Mitok* deletion did not affect muscle structure as demonstrated by hematoxylin and eosin staining. Scale bar 50 µm. **B** *Mito*K deletion did not affect fibre size either of glycolytic (EDL) or oxidative (soleus) muscles. t test (two-tailed, unpaired) of five animals per condition. Data are presented as mean ± SD. **C** Mean maximal running time in a single 10° uphill bout of run on a treadmill of WT and *Mitok*^*−/−*^ mice indicated that *Mitok* deletion negatively affects exercise performance. *p < 0.05, t test (two-tailed, unpaired) of five animals per condition. Data are presented as mean ± SD. **D** Mean maximal running time in a single 10° downhill bout of run on a treadmill of WT and *Mitok*^*−/−*^ mice indicated that *Mitok* deletion negatively affects exercise performance. *p < 0.05, t test (two-tailed, unpaired) of five animals per condition. Data are presented as mean ± SD. **E** Force production of plantar flexors in vivo after electrical stimulation of the nerve normalized for muscle weight. No change in either twitch (4 Hz) or tetanic (100–150 Hz) force in *Mitok*^*−/−*^ animals was measured. Data are presented as mean ± SEM. **F**. Reduction of isometric force production during repeated eccentric contractions shows an increase in force reduction in *Mitok*^*−/−*^ animals. Contractions were performed every 20 seconds to avoid force reduction due to fatigue. *p < 0.05, t test (two-tailed, unpaired) of five animals per condition. Data are presented as mean ± SEM.

In *Mitok*^−/−^ muscles neither signs of damage were detected (Fig. 2A), nor differences in fibre size were observed, both in fast-twitch (i.e. extensor digitorum longus, EDL) and in slow-twitch (i.e. soleus) muscles (Fig. 2B), suggesting that MITOK ablation does not alter the overall histological features of the skeletal muscle.

Next, we wished to know whether mitoK_ATP_ was required for muscle activity. We detected an impaired running capacity on a treadmill until exhaustion, both uphill (Fig. 2C) and downhill (Fig. 2D), indicating that MITOK was required to sustain endurance exercise. We then performed in vivo measurements to assess the force of hindlimb muscles when subjected to contractions at increasing frequency until tetanus is reached. No difference was observed between the two genotypes, indicating that mitoK_ATP_-dependent mitochondrial K^+^ entry was unnecessary to sustain a single bout of tetanic force production (Fig. 2E). During exercise, muscles are also subjected to the lengthening effects of eccentric contractions, that cause sarcolemma damage progressively decreasing force production. The role of mitochondrial K^+^ homeostasis was assessed by measuring in vivo the residual tetanic force of the hindlimb upon repetitive lengthening contractions (Fig. 2F). This assay highlighted the increased force reduction of *Mitok*^*−/−*^ mice compared to WT controls when subjected to the tearing effects of lengthening contractions, indicating that physiological mitochondria K^+^ entry is required to counteract muscle damage.

### MITOK overexpression alters skeletal muscle mitochondrial structure and function

**Fig. 3:**
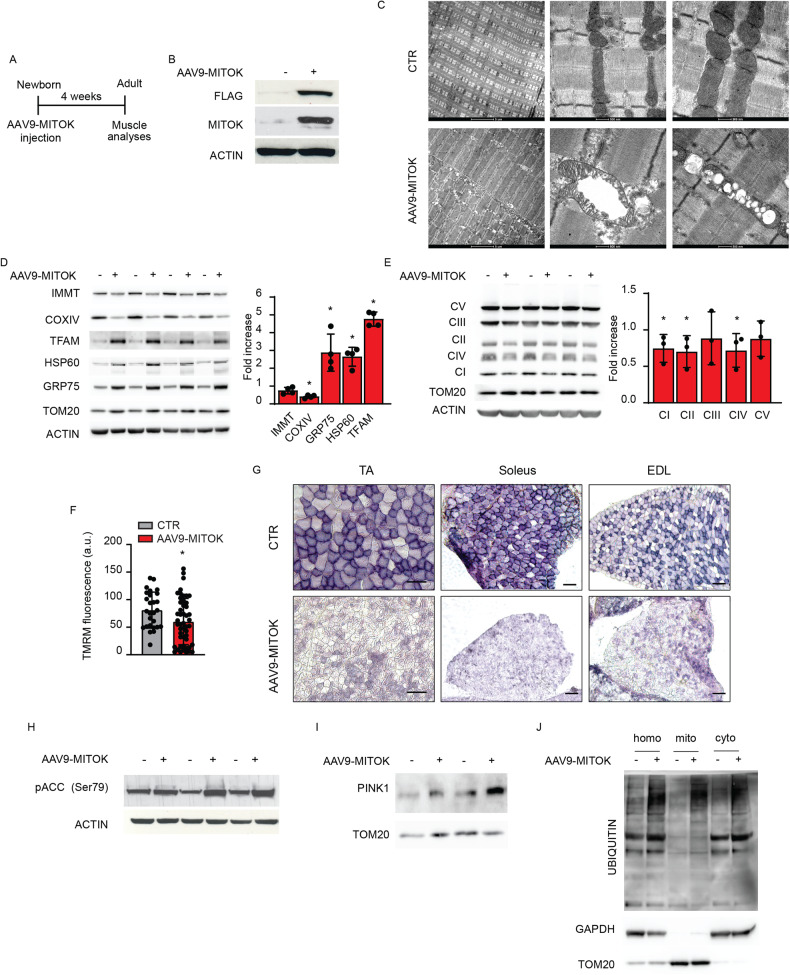
Skeletal muscle MITOK overexpression triggers a severe impairment of mitochondrial morphology. **A** Representative scheme of the experimental design. **B** Hindlimb muscles of newborn mice (4–6 days old) were injected with AAV9-MITOK or control virus. One month later muscles were isolated and processed. Western blot analysis demonstrated increased MITOK protein levels in AAV9-MITOK injected TA muscles. ACTIN was used as protein loading control. **C** TEM analysis performed on the longitudinal section of MITOK-overexpressing TA muscles and control muscles. Pictures shows the presence of swollen mitochondria deprived of internal cristae. **D**. Western blot analyses of AAV9-MITOK infected TA muscles showed alteration in mitochondrial protein levels. ACTIN and TOM20 were used as protein loading control. On the right: quantification by densitometry. Data are reported as fold increase of each protein, normalized for the relative ACTIN compared to control. *p < 0,05, **p < 0,01, t test (two-tailed, unpaired) of four muscles per condition. Data are presented as mean ± SD. **E** Western blot analyses of ETC components in TA muscle overexpressing MITOK and control muscles. ACTIN and TOM20 were used as protein-loading control. On the right: quantification by densitometry. Data are reported as fold increase of each protein, normalized for the relative ACTIN compared to control. *p < 0,05, t test (two-tailed, unpaired) of three animals per condition. Data are presented as mean ± SD. **F** FDB fibres were loaded with TMRM and Δψm was measured. *p < 0,05 t test (two-tailed, unpaired) of at least 25 fibres per condition. Data are presented as mean ± SD. **G** SDH staining performed on TA, soleus, EDL muscles showed reduced SDH-positive fibres in MITOK-overexpressing muscles. Scale bar 100 µm. **H** Western blot analyses demonstrated increased phosphorylation levels of ACC in MITOK-overexpressing muscles compared to control. ACTIN was used as protein-loading control. **I** Western blot analyses demonstrated PINK1 accumulation in MITOK-overexpressing muscles compared to control. TOM20 was used as protein-loading control. **J** Immunoblotting analyses showed increased ubiquitination levels in MITOK-overexpressing muscles compared to controls in the total homogenate and in the different subcellular fractions (mitochondria and cytosol). GAPDH and TOM20 were used as subcellular fractionation controls.

In HeLa cells, MITOK overexpression causes mitochondrial fragmentation, swelling and collapse of cristae [5]. To investigate the effects of MITOK overexpression on mitochondrial morphology and function in skeletal muscle during postnatal development, we injected hindlimbs of newborn mice with adeno-associated viral particles overexpressing Flag-tagged MITOK (AAV9-MITOK). Muscles were collected and analyzed 4 weeks later (Fig. 3A), and western blot analysis confirmed the expression of AAV9-MITOK (Fig. 3B, S4).

Electron microscopy images of longitudinal sections of EDL muscles revealed that MITOK overexpression strongly affects mitochondrial morphology. Many mitochondria were swollen, and internal cristae were collapsed (Fig. 3C), as previously observed in HeLa cells, in which the constitutive opening of the mitoK_ATP_ channel triggers a net influx of potassium cations leading to water entry, subsequent mitochondrial swelling and accumulation of aberrant mitochondria [5]. In line with the alteration of mitochondrial morphology, immunoblot analysis of mitochondrial proteins upon MITOK overexpression suggested a decreased content of proteins localized at the inner mitochondrial membrane, including IMMT and COXIV. We also observed an enrichment in GRP75, HSP60, and TFAM, which mainly localize into the matrix, possibly due to expression induction or decreased degradation for reasons that may be related to cell stress activation pathways (Fig. 3D, S4).

Analysis of ETC components revealed that MITOK overexpression significantly decreases the expression of the subunit NDUFB8 of NADH Dehydrogenase complex I, of the SDHB subunit of complex II, and of the subunit I of complex IV (Fig. 3E, S4). We further evaluated the consequence of the mitochondria structural defects exerted by MITOK overexpression by assessing ΔΨm, that was decreased in AAV-MITOK infected myofibres compared to controls (Fig. 3F). Moreover, we determined SDH activity on tibialis anterior (TA), EDL, and soleus muscle cryosections. The number of SDH activity-positive myofibres in MITOK-overexpressing muscles strongly decreased compared to control muscles (Fig. 3G). The negative impact of MITOK overexpression on mitochondrial function was also revealed by the increased phosphorylation of acetyl CoA carboxylase (ACC), a target of the energy stress sensor AMPK that is activated by a decrease in the energy charge triggering catabolic responses (Fig. 3H, S4). Thus, a bioenergetic defect occurs in consequence of dysregulated mitochondrial K^+^ entry and organelle structure alterations.

The mitochondrial dysfunction of MITOK-overexpressing muscles prompted us to determine the involvement of the PINK1-Parkin pathway, which participates in the response to mitochondria stress. PINK1, which is continuously degraded in physiological conditions, accumulates on the mitochondrial outer membrane (OMM) upon IMM depolarization and mitochondrial damage. On the mitochondrial surface, PINK1 recruits, phosphorylates, and thus activates, the E3 ubiquitin ligase Parkin, which triggers polyubiquitination of several targets at the OMM, priming the selective removal of aberrant organelles by mitophagy. Thus, we thought of determining whether PINK1 accumulates upon MITOK overexpression, and whether Parkin is activated. We observed increased PINK1 protein levels in MITOK-overexpressing muscles compared to controls (Fig. 3I, S4). However, assessment of Parkin recruitment to mitochondria was hampered by the fact that MITOK overexpression alters Parkin protein levels (Figure S2A, S5). Nonetheless, the mitochondrial fraction of MITOK-overexpressing muscles was enriched in polyubiquitinated proteins, indicating the accumulation of ubiquitin-targeted mitochondria (Fig. 3J, S4). Moreover, general activation of the ubiquitin-proteasome system occurs, as demonstrated by the accumulation of polyubiquitinated proteins in the total cell lysates.

Overall, these data indicate that MITOK overexpression induces a drastic alteration of mitochondrial ultrastructure, a decrease of mitochondrial function, and activation of mitochondrial damage-sensing pathways.

### MITOK overexpression impinges on skeletal muscle catabolism and stress response programs

Damaged or dysfunctional mitochondria lead to defective energy production and altered signaling pathways impinging on nuclear gene programs that regulate muscle mass [11]. Accordingly, mitochondria quality control pathways play a central role in regulating the cell programs that underlie muscle homeostasis. In this context, the accumulation of polyubiquitinated proteins at the OMM represents a signaling event for the selective removal of aberrant mitochondria and, in this process, both proteasome and autophagy pathways are involved.

**Fig. 4:**
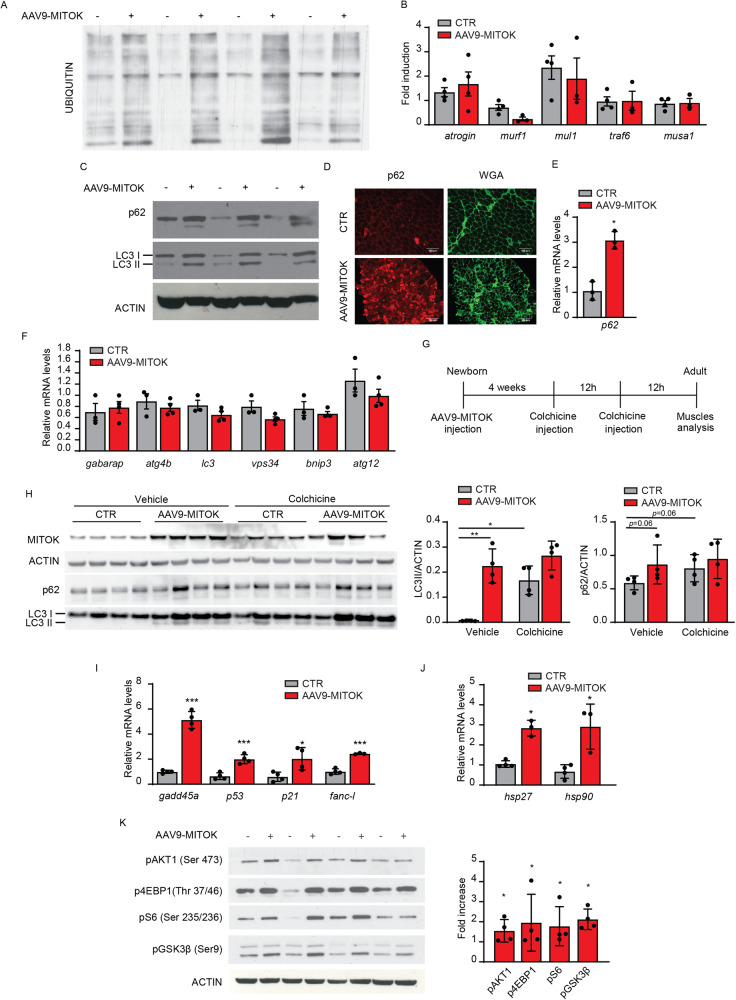
MITOK overexpression impinges on skeletal muscle catabolism and stress response programs. **A** Immunoblotting analyses showed increased ubiquitination levels in MITOK-overexpressing muscles compared to controls. **B.** No difference in ubiquitin ligases gene expression was detected by qPCR analyses. Expression levels are normalized for actin. t test (two-tailed, unpaired) of four animals per condition. Data are presented as mean ± SD. **C** Western blot analyses demonstrated increased p62 and LC3II protein levels in MITOK-overexpressing muscles compared to control. ACTIN was used as protein-loading control. **D** Immunofluorescence analysis performed on transversal cryosections of soleus muscles overexpressing MITOK showed enhanced p62 signal. Scale bar 100 micron. **E**. MITOK overexpression induced the expression of *p62*. Expression levels are normalized for *actin*. *p < 0.05, t test (two-tailed, unpaired) of three animals per condition. Data are presented as mean ± SD. **F** No difference in autophagy-related genes was detected in MITOK-overexpressing muscles compared to controls by qPCR analysis. Expression levels are normalized for *actin*. t-test (two-tailed, unpaired) of at least 3 muscles per group. Data are presented as mean ± SD. **G** Hindlimb muscles of newborn mice were injected with AAV9-MITOK. One month later, mice were treated with colchicine twice with 12 h interval. 12 h after the second injection muscles were analyzed to measure the autophagy flux. **H** Protein levels of LC3-II and p62 were used to monitor autophagy flux, relative to actin protein levels used as loading control. On the right: quantification by densitometry of the ratio between LC3-II/ACTIN and p62/ACTIN. *p < 0,05; **p < 0,01; one-way ANOVA of four animals per condition. Data are presented as mean ± SD. **I** MITOK-overexpressing muscles showed increased expression of genes involved in the stress response. Expression levels are normalized for *actin*. *p < 0.05, ***p < 0.001, t test (two-tailed, unpaired) of four animals per condition. Data are presented as mean ± SD**. J** MITOK overexpression induced the expression of HSPs. Expression levels are normalized for *actin*. *p < 0.05, ***p < 0.001, t test (two-tailed, unpaired) of four animals per condition. Data are presented as mean ± SD. **K** Western blot analyses of AAV9-MITOK muscles demonstrated AKT pathway activation. On the right: quantification by densitometry. Data are reported as fold increase of each protein, normalized for the relative ACTIN compared to control.*p < 0,05; t test (two-tailed, unpaired) of four animals per condition Data are presented as mean ± SD.

Thus, we first thought of verifying the activity levels of the ubiquitin-proteasome system, which plays a key role in muscle atrophy [12]. As anticipated in Fig. 3J, total protein ubiquitination was increased in MITOK-overexpressing muscles (Fig. 4A, S5). However, the expression of muscle-specific E3 ubiquitin ligases, including Atrogin1, Murf1, Mul1, Traf6, and MUSA1, was unaffected (Fig. 4B), suggesting that a steady-state activation level was reached.

We next checked the activity of the autophagy-lysosome system, which on one side is required for continuous turnover of aberrant proteins and organelles, maintaining muscle homeostasis, on the other hand is activated upon catabolic signals [12]. The protein levels of the autophagy marker LC3-II were increased in MITOK-overexpressing TA muscles compared to controls (Fig. 4C, S5). The protein levels of p62/SQSTM1, which is an autophagy receptor bridging polyubiquitinated proteins of the outer membrane of depolarized mitochondria with LC3 on the autophagosome surface, were also increased in MITOK overexpressing muscles (Fig. 4C-D, S5), and this was accompanied by an increase in p62/SQSTM1 transcription (Fig. 4E). However, the mRNA levels of other autophagy genes were unaltered (Fig. 4F), suggesting that the increase in LC3-II levels could be due to hampered autophagosome-lysosome fusion and degradation rather than to autophagy induction. To discern between these two possibilities, we measured the autophagy flux in MITOK-overexpressing muscles compared to controls. As illustrated in Fig. 4G, hindlimbs of newborn mice were injected with AAV9-MITOK or control viral particles. Four weeks later mice were i.p. injected with colchicine twice at 12 h interval, to block autophagosome-lysosome fusion, and sacrificed 12 h after the second injection. As expected, in control muscles colchicine triggered the accumulation of LC3-II indicating an increase in the number of autophagosomes due to a block in their degradation (Fig. 4H). The levels of the p62 protein demonstrated a tendency to rise as well (Fig. 4H).

In MITOK overexpressing muscles either treated with colchicine or not, LC3-II and p62 levels were comparable, indicating that MITOK overexpression causes a block in the autophagic flux (Fig. 4H).

The accumulation of dysfunctional mitochondria and the impairment in their removal is causative of cell stress activation. In MITOK overexpressing muscles, different genes implicated in cellular stress response and DNA repair were induced, including growth arrest and DNA damage-inducible 45α (GADD45a), which is upregulated by fasting and immobilization-induced atrophy [13, 14], p53 and its downstream target p21, both involved in muscle atrophy [15, 16], and FANCL, one of the genes of the Fanconi Anemia (FA) family, required for the activation of the FA pathway, that is involved in DNA repair [17] (Fig. 4I). In addition, heat shock proteins HSP27 and HSP90, that play a fundamental role in the maintenance of muscle homeostasis [18], were also induced (Fig. 4J).

Thus, mitochondrial dysfunction, altered quality control systems and catabolic pathways, and a cell-stress response characterize MITOK-overexpressing muscles. We thought of further investigating muscle protein regulation in conditions of constitutive mitochondrial K^+^ entry by monitoring the IGF1/AKT1 pathway, which controls muscle mass both by inducing protein synthesis and by inhibiting protein degradation. In AAV9-MITOK injected mice, AKT1 pathway was activated, as demonstrated by the increase in AKT1 phosphorylation and of its downstream effectors (Fig. 4K, S5). In particular, MITOK-overexpressing muscles showed an increase in the phosphorylation of 4EBP1, an inhibitor of protein translation, inactivated by AKT-dependent phosphorylation. The phosphorylated form of S6, a positive regulator of protein translation, also increased in MITOK-overexpressing muscles. Finally, GSK3β, a positive regulator of protein synthesis and AKT target was also hyperphosphorylated in MITOK-overexpressing muscles, suggesting that protein synthesis is active.

### MITOK overexpression causes muscle mass loss

**Fig. 5:**
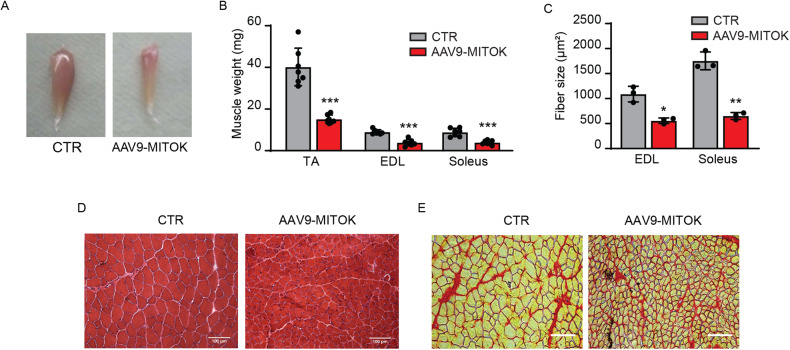
Skeletal muscle MITOK overexpression affects muscle mass. **A** Representative pictures show mass reduction in TA muscle 4 weeks after AAV9-MITOK infection compared to control. **B** TA, EDL and soleus muscles 4 weeks after AAV9-MITOK injection showed a decrease in muscle weight. ***p < 0.001, t test (two-tailed, unpaired) of eight animals per condition. Data are presented as mean ± SD. **C** EDL and soleus muscles fibre size was decreased in AAV9-MITOK injected animals. *p < 0,05; **p < 0,01; t test (two-tailed, unpaired) of three animals per condition. Data are presented as mean ± SD. **D** Hematoxylin and eosin staining showed atrophic fibres in MITOK overexpressing TA muscles 4 weeks after infection. Scale bar 100 µm. **E** No differences were detected by Sirius red staining performed on EDL muscles of AAV9-MITOK injected animals compared to controls. Scale bar 100 µm.

The complex scenario comprising altered mitochondrial function, activation of mitophagy-related pathways in concert with block of the autophagic flux, and induction of AKT1 pathway, prompt the investigation of the effects of constitutive mitoK_ATP_ opening on muscle mass and architecture. We analyzed muscles both four (Fig. 5) and eight (Figure S3A) weeks after AAV9-MITOK infection to evaluate the progression of the phenotype. MITOK overexpression negatively affected muscle size during post-natal growth. In particular, the reduction in muscle weight of AAV9-MITOK injected muscles was more than 50% both 4 and 8 weeks after the infection (Fig. 5A-B, S3B), which was due to great decrease of the mean fibre area compared to controls (Fig. 5C, S3C). Additionally, as demonstrated by H&E (Fig. 5D) and Sirius red (Fig. 5E, S3D) staining, fibrotic area were not detected, suggesting that MITOK overexpression triggers muscle atrophy as a consequence of a specific reduction in fibre size.

### MITOK overexpression in adulthood triggers muscle atrophy

**Fig. 6:**
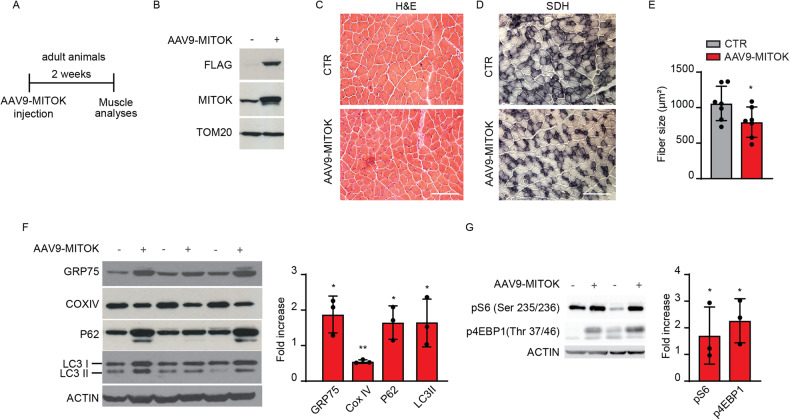
MITOK overexpression during adulthood triggers muscle atrophy. **A** Representative scheme of the experimental design. **B** EDL muscles of 2-month-old mice were injected with AAV9-MITOK or control virus. Two weeks later muscles were isolated and processed for further analyses. Western blot analyses demonstrated increased MITOK protein levels in AAV9-MITOK injected EDL muscles. TOM20 was used as protein loading control. **C** H&E staining revealed no signs of degeneration in MITOK-overexpressing muscles. **D** SDH staining showed no differences in MITOK-overexpressing muscles compared to WT. **E** EDL fibre size was decreased in AAV9-MITOK injected animals. *p < 0,05; t test (two-tailed, unpaired) of three animals per condition. Data are presented as mean ± SD. **F** Western blot analyses of AAV9-MITOK infected muscles showed altered mitochondrial and autophagy protein levels. ACTIN was used as protein loading control. On the right: quantification by densitometry. Data are reported as fold increase of each protein, normalized for the relative ACTIN, compared to control. *p < 0,05, **p < 0,01, t test (two-tailed, unpaired) of three muscles per condition. Data are presented as mean ± SD. **G** Western blot analyses of AAV9-MITOK muscles demonstrated AKT pathway activation. On the right: quantification by densitometry. Data are reported as fold increase of each protein, normalized for the relative ACTIN, compared to control. *p < 0,05; t test (two-tailed, unpaired) of three animals per condition. Data are presented as mean ± SD.

Postnatal growth of skeletal muscle relies on both protein synthesis and myonuclear accretion. During adulthood, myofibre size depends on protein synthesis/degradation balance, while myonuclear number remains mainly constant [19, 20]. We wished to understand whether the effects of MITOK overexpression are limited to the growing muscle or rather are detectable also when mitoK_ATP_ is constitutively open exclusively during adulthood. To this aim, EDL muscles of adult mice were infected with AAV9-MITOK and analyzed 2 weeks later (Fig. 6A). MITOK expression was confirmed by western blot analyses (Fig. 6B, S5). In adult muscles upon MITOK overexpression neither signs of degeneration were present (Fig. 6C), nor SDH staining indicated major mitochondrial dysfunction (Fig. 6D). However, fibre size was decreased in MITOK-overexpressing muscles compared to controls, although less than in muscles injected with AAV9-MITOK soon after birth (Fig. 6E). In addition, similarly to post-natal overexpression, MITOK in adulthood altered the expression of mitochondrial markers and caused an increase in p62 and LC3-II protein levels (Fig. 6F, S5). Finally, MITOK overexpression in the adult muscle triggered the activation of the AKT1 pathway, including phosphorylation of S6 and 4EBP1 (Fig. 6G, S5). In conclusion, MITOK overexpression in adulthood exerted similar effects compared to post-natal growth, including atrophy, alterations of mitochondria and autophagy protein expression, and activation of AKT1 pathway, indicating that the myofibre is the primary target of the increased mitochondrial K^+^ entry.

## Discussion

This represents the first account of the effects of mitoK_ATP_ deletion, and thus of impaired mitochondrial K^+^ fluxes in the living animal. As previously reported, mitoK_ATP_ opening elicits cardioprotection in ischemia/reperfusion injury, and this effect is lost in the hearts of *Mitok*^−/−^ mice. *Mitok*^*-/-*^ animals are otherwise viable and fertile, with no overt phenotype. Here, we aimed at clarifying whether skeletal muscle is susceptible to the effects of *Mitok* deletion previously observed in HeLa cells, including altered mitochondrial cristae width and metabolism, and whether loss of mitochondrial K^+^ homeostasis exerts any consequence on muscle structure and activity. Skeletal muscle mitochondria were as susceptible as those of cell lines to the widening effects of *Mitok* deletion on mitochondrial cristae, and these data were corroborated by a metabolomic analysis highlighting the role of mitochondria K^+^ homeostasis on phospholipid, sphingolipid, and cholesterol content, suggestive of membrane remodeling, that may negatively affect muscle contraction, as previously suggested [21].

Similarly to what observed in cell lines, the defects in mitochondrial shape were causative of altered oxidative metabolism which, together with membrane remodeling, translated into a reduced running capacity both uphill, testing endurance capacity, and downhill, where also damaging lengthening contractions take place. It is important to highlight that the diminished exercise performance observed in *Mitok*^*−/−*^ animals on the treadmill may be linked, at least partially, to the disturbance of mitochondrial K^+^ homeostasis in tissues beyond skeletal muscle, notably in the heart. To gain a clearer understanding of the specific contribution of skeletal muscle to this reduced performance, strength determinations of the hindlimb are instrumental. In vivo force measurements in the anesthetized mouse revealed a major role of mitoK_ATP_ in preserving muscle force upon repetitive lengthening contractions, while it was dispensable for single bouts of tetanic force production. Thus, in agreement with the cytoprotective role of mitoK_ATP_, both downhill running and direct force measurements of the hindlimb highlighted the importance of mitochondrial K^+^ entry during damaging lengthening contractions. On the other hand, the ATP demand occurring during endurance training requires prompt mitoK_ATP_-dependent response, while short-term tetanic force production does not require acute mitochondrial K^+^ entry. These data are in line with the low activity of mitoK_ATP_ channels in the presence of resting levels of cytosolic ATP and unveil the need of mitochondrial volume adjustments to meet the energy requirements of the working muscle. However, it should be noted that adaptations occurring in constitutive knockout animals may hinder phenotypic manifestations that may instead result from acute gene deletion. Moreover, ischemic preconditioning has been observed in skeletal muscle [22]. Thus, whether mitoK_ATP_ plays a role in the protection of skeletal muscle during hypoxia and reoxygenation that may occur during muscle work remains to be established.

At the opposite side, MITOK overexpression triggers mitochondrial swelling and loss of membrane potential, thus representing an unvaluable genetic tool to study the effects of mitochondrial poisoning in a specific and tightly controlled manner. MITOK overexpression alters the organelle architecture in muscle and causes energy default with detrimental effects on organ physiology, highlighted by the dramatic atrophy. As for the signaling pathways involved, mitochondrial stress triggered the accumulation of PINK1 and of polyubiquitinated proteins at mitochondria, and the activation of the energy sensor AMPK. Mitochondria dysfunction and polyubiquitination of OMM proteins are signals for the recruitment of autophagy receptors and, together with AMPK induction, are supposed to additionally induce macroautophagy, the degradative system responsible for the removal of bulk aberrant proteins and organelles. However, no induction of autophagy-related genes is detected in MITOK overexpressing muscles. At the opposite, the autophagy flux is blocked, determining the accumulation of undigested material and aberrant mitochondria, and further contributing to atrophy in a vicious cycle. Given that autophagy operates continuously within the cell to generate metabolites crucial for sustaining cellular metabolism, eliminate damaged components, and enhance repair and stress resistance, any disruption in autophagy can lead to muscle degeneration and weakness [12]. Autophagy deficiency is indeed per se sufficient to trigger loss of muscle mass, as demonstrated by the atrophic phenotype of muscles depleted of the essential autophagy gene Atg7 [23]. Additionally, the Akt pathway that sustains protein synthesis and hampers protein degradation, is induced. Akt inhibits autophagosome formation, suggesting that, in MITOK-overexpressing muscles, Akt activation most likely represents a compensatory response, still frustrated, to the detrimental effects of excessive mitochondrial K^+^ entry. Moreover, polyubiquitinated proteins are increased, suggesting both an accumulation due to the defective autophagic process, and an augmented delivery of aberrant proteins to the proteasome system. However, mRNA levels of muscle-specific ubiquitin ligases are unchanged, indicating that a steady-state response to the long-term mitochondrial damage is reached. Thus, excessive accumulation of K^+^ in mitochondria results in organelle dysfunction and disruption of the main degradative systems necessary for maintaining mitochondrial health. Similarly, an imbalance in mitochondrial quality control pathways leads to impaired mitophagy and the buildup of malfunctioning organelles [11]. Therefore, the overexpression of MITOK initiates a conserved catabolic program.

This scenario also involves activation of a cell-stress response transcriptional program. GADD45a is a nuclear protein that has been described as a stress sensor implicated in the regulation of many cellular functions including DNA repair, cell cycle arrest and apoptosis. In skeletal muscle, GADD45a expression is increased by fasting [13] and immobilization [14] and is required for atrophy induced by different stimuli [14]. In MITOK-overexpressing muscles, GADD45a mRNA levels were increased, indicating that it may play a primary role. Various mechanisms may be responsible for GADD45a induction. For example, heterodimers of ATF4 with C/EBPβ mediate muscle atrophy by binding and activating an evolutionary-conserved regulatory element in the Gadd45a gene [24]. Moreover, HDAC4 is upstream of GADD45a in denervation-induced atrophy [25], and additional undetermined factors are also involved.

In addition, p53, p21, FANC-L, and the HSP genes HSP27 and HSP90, involved in growth suppression, DNA repair or stress-response pathways in skeletal muscle, were upregulated, suggesting a coordinated transcriptional response to the primary atrophy trigger. In skeletal muscle, p53 activates muscle atrophy, while loss of p53 expression partially protects from immobilization-induced atrophy [16]. p21 promotes atrophy by reducing the expression of spermine oxidase, which is involved in the maintenance of muscle fibre size in physiological conditions [15]. Finally, HSPs represent an important skeletal muscle defense system induced by a variety of environmental burdens such as oxidative stress, inflammation and damage [18]. Thus, the gene expression profile induced by MITOK overexpression points to a multi-faceted stress-response pattern.

In summary, mitoK_ATP_ activity controls skeletal muscle mitochondria volume and cristae shape, impinging on metabolism and energy production. Accordingly, loss of ATP-regulated mitochondrial K^+^ entry translates into defective muscle activity, loss of cytoprotection and increased sensitivity to damaging triggers. On the other side, uncontrolled K^+^ entry by overexpression of MITOK leads to the dissipation of the electrochemical gradient for protons, resulting in mitochondrial damage and triggering a cellular response that involves the activation of specific signaling pathways responsible for muscle mass loss. Overall, the fine-tuned modulation of mitoK_ATP_ activity is required for the maintenance of skeletal muscle homeostasis and function. Thus, our study not only validates prior evidence regarding the role of mitoK_ATP_ in regulating mitochondrial volume but also underscores the function of mitoK_ATP_-dependent potassium flux in skeletal muscle pathophysiology. Further studies are required to determine the regulation of Mitok_ATP_ activity in diverse atrophy conditions, such as disuse, denervation, cachexia, or myopathies—whether of mitochondrial origin or seemingly unrelated. Additionally, a comprehensive exploration is warranted to determine if modulating MitoK_ATP_ could serve as a therapeutic target in these conditions. Furthermore, future investigations are necessary to unravel the intricate interplay between mitoK_ATP_ and other mitochondrial potassium channels in various scenarios of skeletal muscle activity and disease. This includes elucidating whether their activities synergize in controlling potassium fluxes and understanding the mechanisms of their modulation.

## Materials and methods

### Animals

*Mitok*^*−/−*^ mice were generated by genOway on a C57BL/6 N background. Two LoxP sites flanking exon 4 of the mouse *Mitok* gene were introduced by homologous recombination. The genotype was verified by PCR with the following primers:

Mitok knockout fw 1 GCACCTTGTCAGCACCATGACAACTC

Mitok knockout fw 2 GAGGGATCGCTGTGGAAGGCTGTAT

Mitok knockout rv GCGGACAAAGATTGTGTCACTGTTTGC

The knockout allele yields an amplification product of 769 bp, whereas the wild-type allele generates a 278-bp fragment. CD1 mice was provided by Charles River. All mouse experiments were performed in accordance with the Italian law D. L.vo n°26/2014.

### Metabolomics analysis

Gastrocnemius muscles of adult mice were harvested and underwent untargeted metabolomics analysis performed by Metabolon, Inc. Briefly, samples were prepared using the automated MicroLab STAR® system from Hamilton Company. The resulting extract was divided into five fractions: two for analysis by two separate reverse phase (RP)/ultra-performance liquid chromatography tandem mass spectrometry (UPLC-MS/MS) methods with positive ion mode electrospray ionization (ESI), one for analysis by RP/UPLC-MS/MS with negative ion mode ESI, one for analysis by hydrophilic interaction liquid chromatography/UPLC-MS/MS with negative ion mode ESI, and one sample was reserved for backup.

### Mitochondrial membrane potential (ΔΨm) measurements

FDB Muscles were digested in collagenase A (4 mg/ml) (Roche) dissolved in Tyrode’s salt solution (pH 7.4) (Sigma-Aldrich) containing 10% fetal bovine serum (Thermo Fisher Scientific). Single fibres were isolated, plated on laminin-coated glass coverslips and cultured in Dulbecco’s modified Eagle’s medium (DMEM) supplemented with 25 mM HEPES (Thermo Fisher Scientific), 10% fetal bovine serum, penicillin (100 U/ml) and streptomycin (100 μg/ml). Fibres were maintained at 37 °C in a humidified incubator with 5% CO_2_. After 24 h, fibres were incubated with 20 nM tetramethylrhodamine methyl ester (TMRM, Molecular Probes) in Krebs-Ringer modified buffer (KRB, 135 mM NaCl, 5 mM KCl, 1 mM MgCl_2_, 20 mM HEPES, 1 mM MgSO_4_, 0.4 mM KH_2_PO_4_, 1 mM CaCl_2_, 5.5 mM glucose, pH 7.4) for 30 min at 37 °C. During the experiments, myofibres were maintained in KRB at RT in the presence of 75 μM N-benzyl-P-toluenesulfonamide (BTS, Sigma-Aldrich) to avoid fibre contraction. Images were acquired by confocal microscopy. At the end of each experiment, 10 μM CCCP was added to collapse the ΔΨm. After background correction, the fluorescence value after addition of CCCP was subtracted for each fibre. Analysis was performed by using the Fiji distribution of Image J [26].

### OCR (oxygen consumption rate) measurements

FDB muscles were digested in collagenase A (4 mg/ml) (Roche) dissolved in Tyrode’s salt solution (pH 7.4) (Sigma-Aldrich) containing 10% fetal bovine serum (Thermo Fisher Scientific). Single fibres were isolated, plated on laminin-coated XF24 microplate wells and cultured in DMEM (D5030 Sigma-Aldrich), supplemented with 1 mM NaPyr, 5 mM glucose, 33 mM NaCl, 15 mg phenol red, 25 mM HEPES, and 1 mM of L-Glu. Fibres were maintained for 2 h at 37 °C in 5% CO_2_. The rate of oxygen consumption was assessed in real-time with the XF24 Extracellular Flux Analyzer (Agilent), which allows to measure OCR changes after up to four sequential additions of compounds. A titration with the uncoupler CCCP was performed to utilize the CCCP concentration (0.8 μM) that maximally increases OCR. To calculate basal and maximal respiration, non-mitochondrial O_2_ consumption was subtracted from absolute values. ATP-linked respiration was calculated as the difference between basal and oligomycin-insensitive O_2_ consumption.

The results were normalized for the fluorescence of Calcein (Sigma-Aldrich). Fibres were loaded with 2 μM Calcein for 30 min. Fluorescence was measured using a Perkin Elmer EnVision plate reader in well scan mode using 480/20 nm filter for excitation and 535/20 nm filter for emission.

### Real-time imaging of mitochondrial Ca^2+^ in FDB fibres

FDB muscles were digested in collagenase A (4 mg/ml) (Roche, #COLLA-RO) dissolved in Tyrode’s salt solution (pH 7.4) (Sigma Aldrich, #T2145) containing 10% fetal bovine serum (Thermo Fisher). Single fibres were isolated, plated on laminin-coated glass coverslips, and cultured in DMEM supplemented with 25 mM HEPES (Thermo Fisher, #42430), 10% fetal bovine serum, 100 U/ml penicillin and 100 mg/ml streptomycin (Thermo Fisher, #15070063). Fibres were maintained at 37 °C with 5% CO_2_.

During the experiments, myofibres were maintained in Krebs-Ringer modified buffer (135 mM NaCl, 5 mM KCl, 1 mM MgCl_2_, 20 mM HEPES, 1 mM MgSO_4_, 0.4 mM KH_2_PO_4_, 1 mM CaCl_2_, 5.5 mM glucose, pH 7.4) containing 0.02% pluronic acid for 20 min at 37 °C and then washed with Krebs-Ringer modified buffer in presence of 75 μM N benzyl-P-toluenesulfonamide (Sigma Aldrich, #BL3H160B8B26) to avoid fibre contraction. 30 mM caffeine (Sigma Aldrich, #C0750) was added to elicit Ca^2+^ release from intracellular stores. Experiments were performed on a Zeiss Axiovert 200 microscope equipped with a 40×/1.3 N.A. PlanFluor objective. Excitation was performed with a DeltaRAM V high-speed monochromator (Photon Technology International) equipped with a 75 W xenonarc lamp. Images were captured with a high-sensitivity Evolve 512 Delta EMCCD (Photometrics). The system is controlled by MetaMorph 892 7.5 (Molecular Devices) and was assembled by Crisel Instruments.

To measure mitochondrial Ca^2+^ uptake, fibres were dissected and loaded with 2 μM mt-fura-2.3, an optimized version of the recently developed mitochondria targeted fura-2 Ca^2+^ probe [27]. Images were collected by alternatively exciting the fluorophore at 340 and 380 nm and by recording fluorescence emission through a 515/30 nm band-pass filter (Semrock). Exposure time was set to 100 ms. The acquisition was performed at binning 1 with 200 of EM gain. Image analysis was performed with Fiji distribution of the ImageJ software [26]. Images were background subtracted. Changes in fluorescence (340/380 nm ratio) was expressed as R.

### Mouse treadmill experiments

For acute concentric exercise experiments, 3- or 6-month-old mice were acclimated to and trained on a 10° uphill LE8700 treadmill (Harvard apparatus) for 2 days. On day 1, mice ran for 5 min at 8 m/min and on day 2 mice ran for 5 min at 8 m/min followed by 5 min at 10 m/min. On day 3, mice were subjected to a single bout of running starting at the speed of 10 m/min. Forty minutes later, the treadmill speed was increased at a rate of 1 m/min every 10 min for a total of 30 min and then increased at the rate of 1 m/min every 5 min until mice were exhausted. Exhaustion was defined as the point at which mice spent >5 s on the electric shocker without attempting to resume running. Total running time and total running distance were recorded for each mouse.

For eccentric exercise experiments, 3-month-old mice were acclimated to and trained on a 10° downhill treadmill for 2 days. On day 1, mice ran for 5 min at 8 m/min and on day 2 mice ran for 5 min at 8 m/min followed by 5 min at 10 m/min. On days 3–5, mice were subjected to a single bout of running starting at the speed of 10 m/min. Forty minutes later, the treadmill speed was increased at a rate of 1 m/min every 10 min for a total of 30 min and then increased at the rate of 1 m/min every 5 min until mice were exhausted. Exhaustion was defined as above. Total running time and total running distance were recorded for each mouse.

### Force measurements

Muscle function in vivo was assessed for the gastrocnemius muscle, as described previously [28]. Briefly, mice were anesthetized with a mixture of tiletamine/zolazepam (40 mg/kg) and xilazine (7 mg/kg) and the foot was mounted on a 305B muscle lever system (Aurora Scientific, ON Canada). A lever arm of 2,1 mm was used for all groups, as no major differences in body weight between various groups was observed. Electrodes were then placed on either side of the sciatic nerve, while the common peroneal nerve was cut. The knee was blocked, and an electrical stimulation was applied to the sciatic nerve, inducing the isometric plantar flexion of the foot. The force-frequency curve was obtained by stimulating at increasing frequencies (starting with a single depolarization up to 150 Hz). Force was then normalized to the weight of gastrocnemius and plantaris muscles, as an estimate of specific force. Eccentric contractions were performed by moving the foot backward at a velocity of 40 mm/s while the gastrocnemius was stimulated with a frequency sufficient to induce full tetanic fusion (100 Hz). Contractions were repeated once every 20 seconds to void inducing fatigue. Experimental data were analyzed using a self-compiled program in LabView.

### AAV9-MITOK administration

AAV9-MITOK was purchased from Vector Biolabs. For experiments in the newborn, 10^10^ vg were injected into the hindlimb of 4- to 6-day-old CD1 male mice. Muscles were subsequently analyzed 4- or 8 weeks postinjection as reported in the Result section. An average of 70% of fibres were positive for the AAV infections. For experiments in the adult, CD1 male mice were used. EDL muscles were isolated through a small hindlimb incision, and 10^10^ vg were injected along the muscle length. FDB muscles were injected with 5 ×10^9^ vg. Muscles were analyzed 15 days post-injection.

### Autophagy flux analysis

Autophagic flux analysis was performed as previously described [29] with some modifications. Hindlimbs muscle of newborn mice were injected with AAV9-MITOK. Colchicine was dissolved in water and stored at -20 °C, as a stock solution, at a concentration 4 mg/ml. On the day of treatment, colchicine was diluted to 0.1 mg/ml in water and 0.1 mg/Kg colchicine was i.p injected. Control mice received an equal volume of NaCl 0.9%. The treatment was repeated 12 hours after the first injection. Mice were sacrificed 24 hours after the first injection, TA muscles were harvested and frozen in liquid nitrogen-cooled isopentane.

### RNA extraction, reverse transcription, and quantitative real-time PCR

Total RNA was extracted from TA muscles using SV Total RNA isolation kit (Promega) following the manufacturer’s instructions. The RNA was quantified with Nanodrop (Thermo Fisher Scientific). From 400 ng of total RNA of each sample, complementary DNA was generated with a cDNA synthesis kit (SuperScript II, Thermo Fisher Scientific) and analysed by real-time PCR using IQ5 thermocycler and SYBR green chemistry (Bio-Rad). The primers were designed and analysed with Primer3 [30]. Real-time PCR standard curves were constructed by using serial dilution of cDNAs of the analysed samples, using at least four dilution points and the efficiency of all primer sets was between 85 and 102%. Actin was used as an internal control for cDNA quantification and normalization of the amplified products. Real-time PCR primer sequences were as follow:

Atrogin-1: FW: GCAAACACTGCCACATTCTCTC RV: CTTGAGGGGAAAGTGAGACG MuRF-1: FW: CCTTCCTCTCAAGTGCCAAG RV: CCTCAAGGCCTCTGCTATGT Mul-1: FW: AGGGCATTCTTTCAGAAGCA RV: GGGGTGGAACTTCTCGTACA Traf6: FW: GCAGTGAAAGATGACAGCGTGA REV: TCCCGTAAAGCCATCAAGCA Fanc-L: FW: GCACGCAGGATTGCATTAGG RV: GCTACCACTCAGCTTCATTCC p21: FW: CGGTGTCAGAGTCTAGGGGAA RV: GAACAGGTCGGACATCACCA P53: FW: GGCGTAAACGCTTCGAGATG RV:TCAGGTAGCTGGAGTGAGCCGADD45α: FW: GAAAGTCGCTACATGGATCAGT RV: AAACTTCAGTGCAATTTGGTTC HSP27: FW: TGACCCAGGCTGGAGTAGAA RV: TGGCTCGGGACAACAACATT HSP90: FW: GGAGATTTTCCTCCGCGAGT RV: GTCATGCCAATGCCTGTGTC

### Mitochondria fractionation

TA and EDL muscles (approx. 250 mg of tissue) were minced and homogenized with a motor-driven pestle (20 strokes) in an isotonic buffer (mIB- mitochondrial Isolation Buffer - 250 mM sucrose, 10 mM KCl, 20 mM HEPES, 1 mM EDTA, pH 7.4). The total homogenate was centrifugated at 1000× *g* for 5 min to remove nuclei and entire cells. The supernatant was then centrifuged at 10000×g for 10 min to pellet the crude mitochondrial fraction. The mitochondrial fraction was washed twice with mIB and centrifuged for 10 min at 10000xg after each washing. The second supernatant was further centrifuged at 40,000× *g* for 20 min to remove light membranes and clear the cytosolic fraction. Proteins of the total homogenate, of the mitochondria fraction, and of the cytosolic fraction were extracted in RIPA buffer (150 mM NaCl, 50 mM TRIS, 1% Triton-X100, 0.1% Na-deoxycholate and 0.1% SDS, pH 7.4) and quantified using the BCA Protein Assay Kit (Thermo Fisher Scientific) following the manufacturer instructions. 20 μg of protein were separated by SDS-PAGE, transferred onto nitrocellulose membranes and probed using the indicated antibodies.

### Western blotting and antibodies

To monitor protein levels, frozen muscles were dissolved by means of Qiagen Tissue Lyser and protein extracts were prepared in a buffer containing: 50 mM Tris pH 7.50, 150 mM NaCl, 5 mM MgCl_2_, 1 mM DTT, 10% glycerol, 2% SDS, 1% Triton X-100, Roche Complete Protease Inhibitor Cocktail, 1 mM PMSF, 1 mM NaVO_3_, 5 mM NaF and 3 mM β-glycerophosphate. 40-60 mg of total proteins were loaded, according to BCA quantification. Proteins were separated by SDS-Page electrophoresis, in commercial 4-12% acrylamide gels (Thermo Fisher Scientific) and transferred onto nitrocellulose membranes (Thermo Fisher Scientific) by semidry electrophoretic transfer. Blots were blocked 1 hour at RT with 5% non-fat dry milk (Bio-Rad) in TBS-tween (0.5 M Tris, 1.5 M NaCl, 0.01% Tween) solution and incubated overnight at 4 °C with primary antibodies. Secondary antibodies were incubated 1 h at RT. After each antibody incubation, three washes of 10’ each were performed with TBS-0.01% tween. The following antibodies were used: anti-pAKT1 (1:100 Cell Signalling #4060), anti-Actin (1:10000 Santa Cruz sc-47778, 1:10000 Proteintech 20536-1-AP), anti-LC3B (1:500 Cell Signalling #2775), anti-p62 (1:3000, Sigma P0067), anti-TOM20 (1:20000 Santa Cruz sc-17764, 1:1000 Cell Signaling #42406), anti-MITOK (1:1000 Sigma HPA010980), anti-GRP75 (1:1000, Santa Cruz sc-133137), anti-IMMT (1:1000, Proteintech 10179-1-AP), anti-COXIV (1:1000, Cell Signaling #4844), anti-HSP60 (1:1000, Santa Cruz sc-59567), anti-TFAM (1:1000, Abnova H00007019-B01P), anti-Parkin (1:1000, Santa Cruz sc-32282), anti-polyubiquitin (1:1000, Enzo BLM-PW8810-0400), anti-pS6 (Ser 240/244; 1:1000, Cell Signalling #5364), anti-pGSK3β (Ser9, 1:1000, Cell Signalling #9336), anti-p4EBP1 (Thr 37/46, 1:1000, Cell Signalling #2855), anti-Flag (1:1000 Cell Signalling #14793). Secondary HRP-conjugated antibodies were purchased from Bio-Rad and used at 1:5000 dilution.

To perform the polyubiquitin WB on the subcellular fractions all the procedures were done in the presence of proteasome inhibitors MG132 (10 µM, Merck) and PR619 (1 µM, Merck).

Densitometric analysis was conducted with the Fiji distribution of ImageJ [26]. Initially, the signal intensity ratio between the protein of interest and the protein used as loading control was quantified. Subsequently, the fold change of the treated muscle versus its contralateral counterpart was calculated and plotted.

### Immunofluorescence

For immunohistochemistry analysis and fibre size measurements, AAV9-MITOK injected muscle cryosections were fixed with 4% FA for 20 minutes, quenched with 50 mM NH_4_Cl in phosphate-buffer saline (PBS) and blocked in PBS containing 0.5% BSA and 10% goat serum for 20 min. Sections were then incubated with primary antibodies for 1 h at 37 °C. The following antibodies were used: anti-flag (1:100, Cell Signaling) and anti-p62 (1:100, Sigma). Alexa Fluor 488- or 555-conjugated secondary antibodies (Thermo Fisher Scientific) were used. For the detection of sarcolemma, WGA antibody (1:1000) was used, while Hoechst was used to mark nuclei. Fibre size measurements were performed with the Fiji distribution of ImageJ [26].

### Hematoxylin and eosin staining

Hematoxylin and Eosin (H&E) staining was performed on 20 µm-thick cryosections of TA and EDL muscles. The staining was carried out using the Rapid Frozen Sections H&E staining Kit (Bio-Optica).

### Sirius red staining

Sirius Red staining was performed on 20 µm-thick cryosections of EDL muscles. Sections were incubated overnight at RT in a Bouin Solution (saturated picric acid, 37% formaldehyde, 5% acetic acid). After washing in running water, sections were incubated in a solution of 0.1% Direct Red 80 in saturated picric acid for 1 h at RT. After washing with 2% acetic acid, sections were dehydrated in sequence with 40-50% ethanol, 70% ethanol, 96% ethanol, 100% ethanol, 1:1 ethanol-xylene (100%), 100% xylene. Slides were mounted with Eukitt (Sigma).

### SDH staining

20 µm-thick cryosections of TA, EDL and soleus muscles were stained with SDH incubating solution, prepared by the addition to SDH stock solution (0.2 M Na^+^-succinate, 0.2 M Phosphate buffer pH 7.4) of nitroblue tetrazolium (final concentration 1 mg/mL), for 20 minutes at 37 °C. Sections were rinsed three times in water and assembled with Mowiol.

### Electron microscopy

EDL muscles were fixed with a fixative solution (3.5% glutaraldehyde in 0.1 M NaCaCo buffer, pH 7.2) at RT. Small bundles of fibres were post-fixed in 2% OsO_4_ in the same buffer for 2 h and then block-stained in aqueous saturated uranyl acetate. After dehydration, specimens were embedded in an epoxy resin (Epon 812). Ultrathin sections (approximately 30-40 nm) were stained in 4% uranyl acetate and lead citrate.

### Statistical analysis of data

The sample size was determined based on similar experiments. Depending on the number of expected comparisons per experimental group, the type I error α was set between 0.55% and 5% bilaterally. The required number of animals is the minimum number needed to achieve a test power of 80%. Statistical data are expressed as mean ± SD unless otherwise specified. For comparison between two independent groups, unpaired t tests were used. Adjusted *p < 0.05, **p < 0.01, and ***p < 0.001. For animal studies, randomization was applied, no blinding was done.

### Supplementary information

Supplemental material

## Data Availability

The datasets generated and analysed during the current study are available from the corresponding author on reasonable request.
