# Supplementary material for: The mitochondrial ATP-dependent potassium channel (mitoKATP) controls skeletal muscle structure and function
Source: Cell Death Dis. 2024 Jan 17;15(1):58. doi: 10.1038/s41419-024-06426-x (PMC10794173; doi:10.1038/s41419-024-06426-x)
Supplement: Supplementary file 1 — Supplemental material [file 41419_2024_6426_MOESM1_ESM.pdf]

Supplementary Material

The mitochondrial ATP-dependent potassium channel (mitoK$_{ATP}$) controls skeletal muscle structure and function

Giulia Di Marco, Gaia Gherardi, Agnese De Mario, Ilaria Piazza, Martina Baraldo, Andrea Mattarei, Bert Blaauw, Rosario Rizzuto, Diego De Stefani, Cristina Mammucari

Figure S1

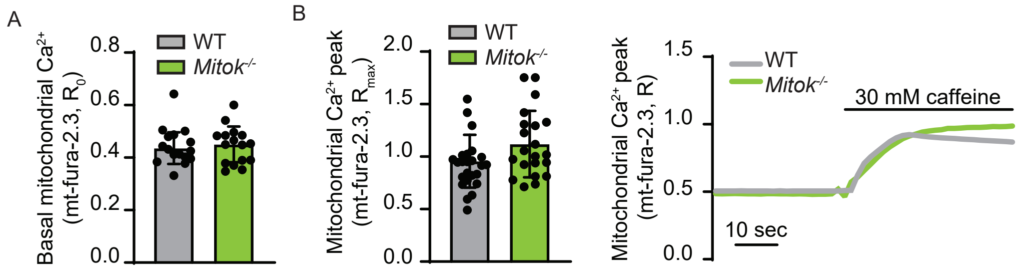

Figure S1.

A. Resting mitochondrial Ca^{2+} levels of single isolated FDB fibres. t test (two-tailed, unpaired) of at least 20 fibres per condition. Data are presented as mean ± SD.

B. Mitochondrial Ca^{2+} uptake in single isolated FDB fibres loaded with mt-fura-2.3. Fibers were stimulated with 30 mM caffeine. On the left: mean mitochondrial Ca^{2+} peaks are shown. On the right: representative traces of mitochondrial Ca^{2+} uptake are shown.

Figure S2

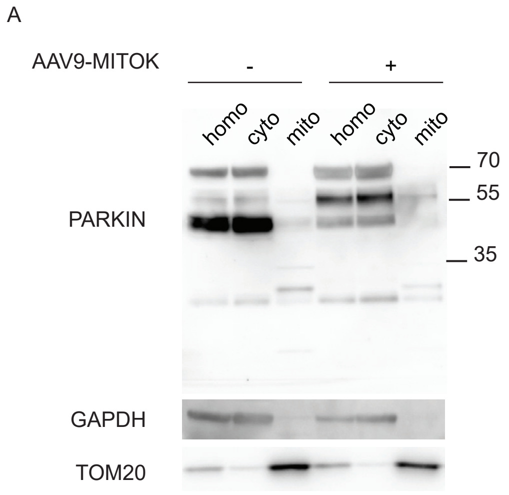

Figure S2.

A.Immunoblotting analysis of PARKIN in MITOK overexpressing muscles compared to controls in the total homogenate and in the different subcellular fractions (cytosol and mitochondria). GAPDH and TOM20 were used as subcellular fractionation controls.

Figure S3

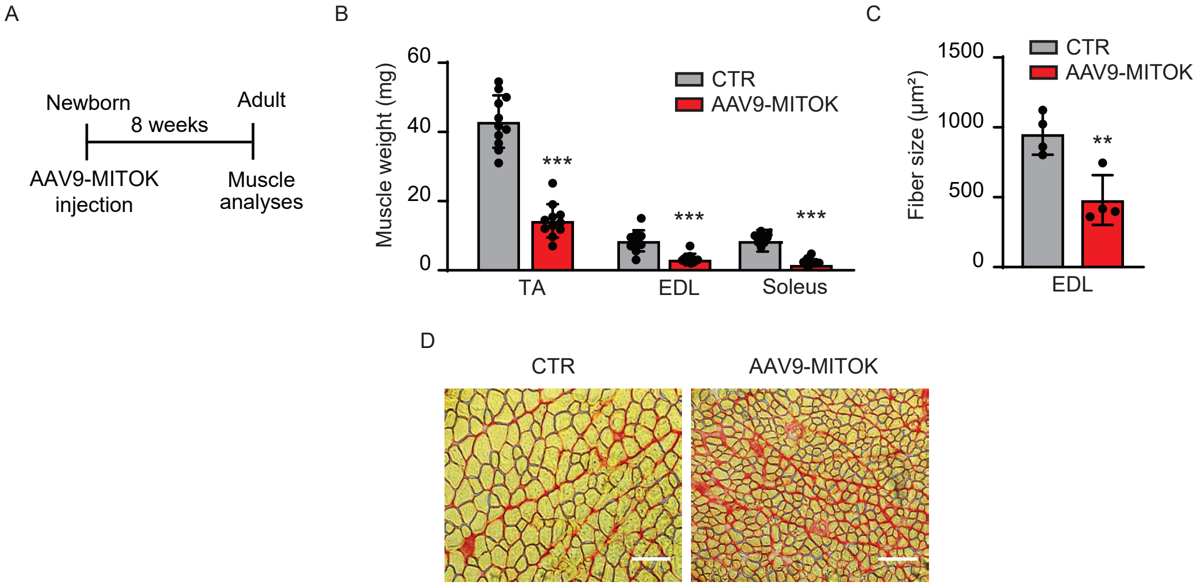

Figure S3.

A. Representative scheme of the experimental design.

B. Hindlimb muscles of newborn mice (4–6 days old) were injected with AAV9-MITOK. Eight weeks later muscles were isolated and processed for further analysis. TA, EDL and soleus muscles show a decrease in muscle weight after AAV9-MITOK injection. ***$p<0.005$, t test (two-tailed, unpaired) of eight animals per condition. Data are presented as mean ±SD.

C. EDL fiber size was decreased in AAV9-MITOK injected animals. **$p<0,01$; t test (two-tailed, unpaired) of three animals per condition. Data are presented as mean ± SD.

D. No differences were detected by Sirius red staining performed on EDL muscles 8 weeks after infection. Scale bar 100 micron.

Figure S4

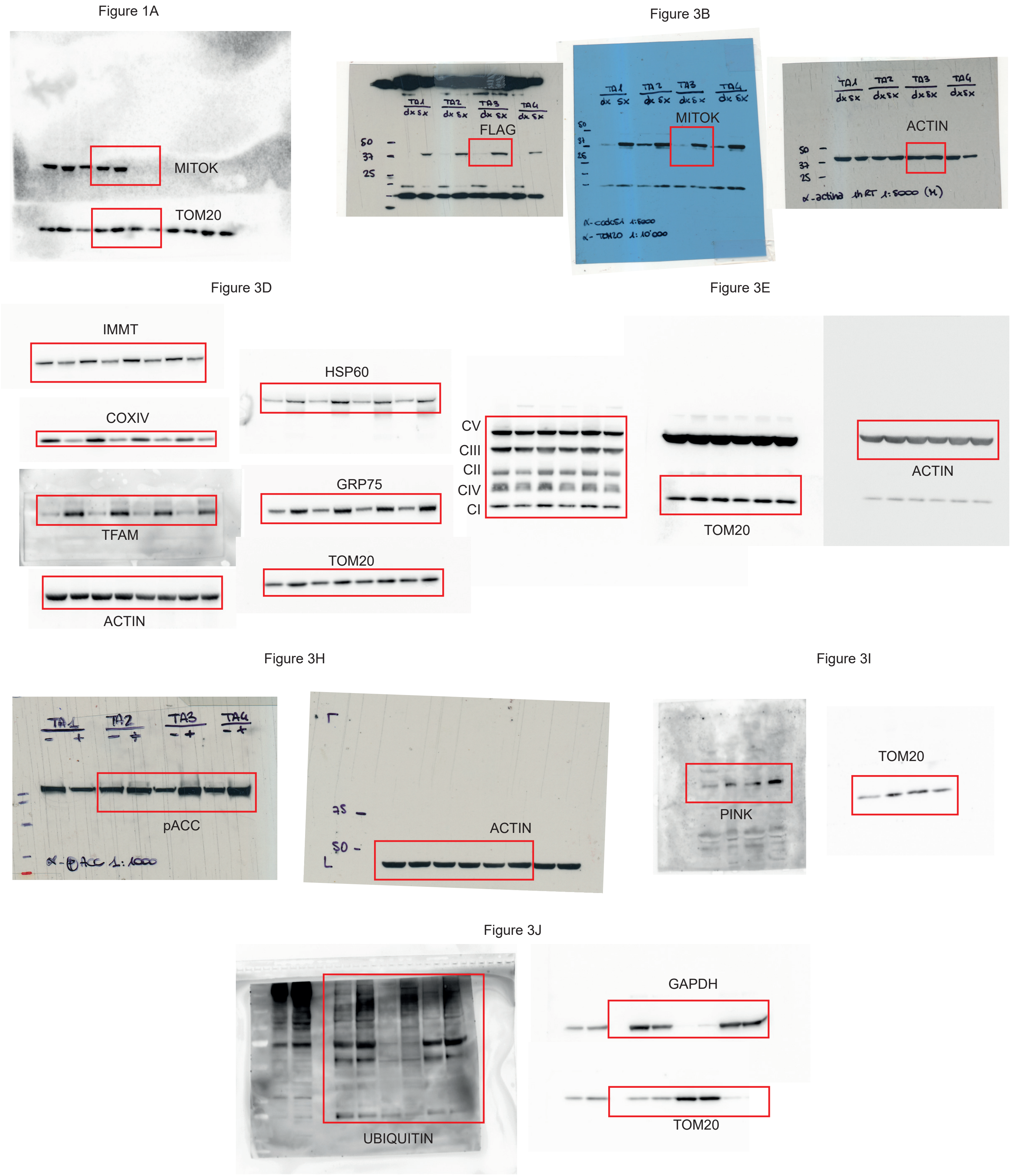

Figure S4. Uncropped images of western blots used in Figures 1 and 3.

Figure S5

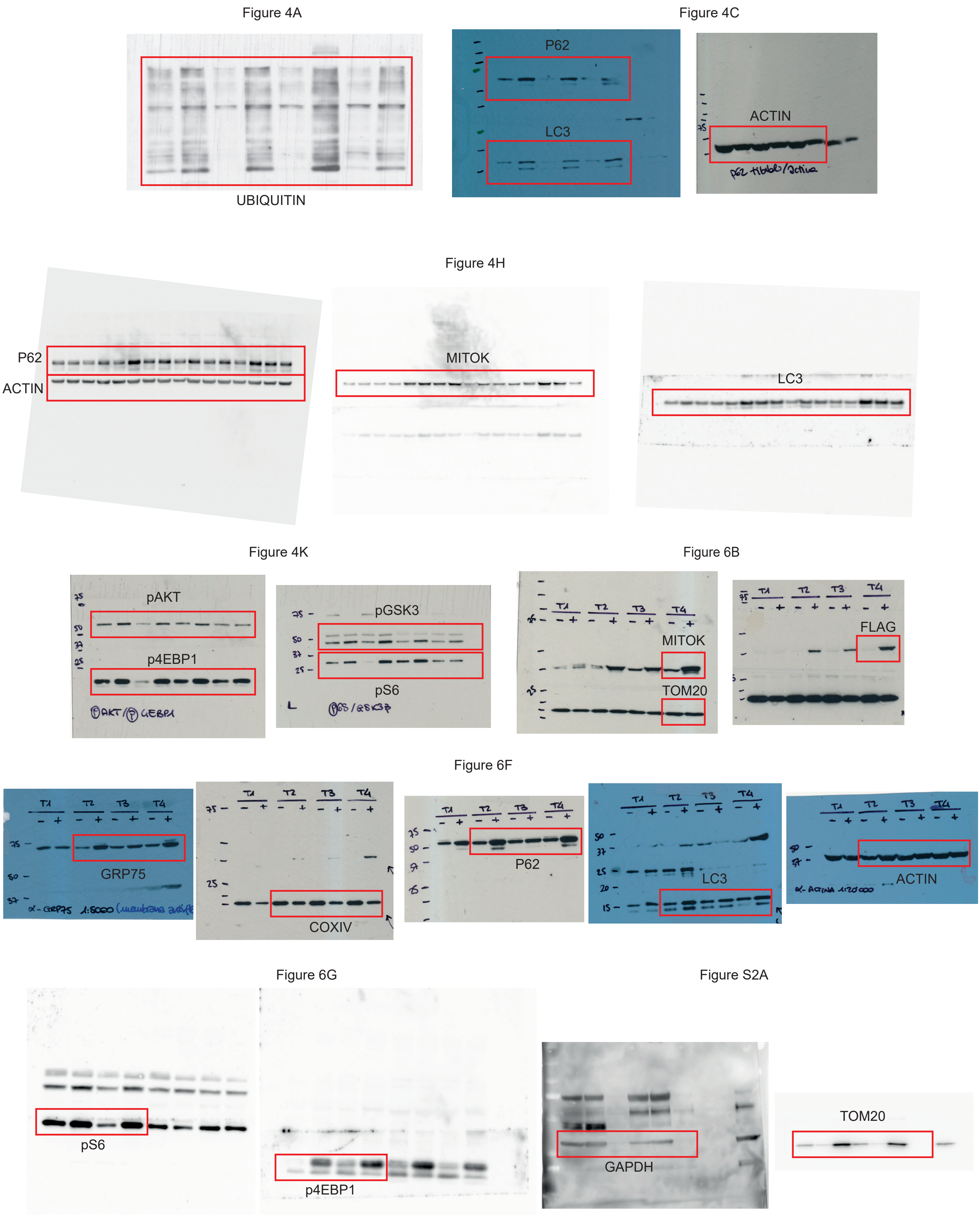

Figure S5. Uncropped images of western blots used in Figures 4, 6, and S2.
